# The epigenetic clock: a molecular crystal ball for human aging?

**DOI:** 10.18632/aging.101712

**Published:** 2019-01-21

**Authors:** Simone Ecker, Stephan Beck

**Affiliations:** 1 UCL Cancer Institute, University College London, London, UK; 2 UCL Institute of Digital Health, University College London, London, UK; 3 UCL Institute for Precision Medicine, University College London, London, UK

**Keywords:** DNA methylation, aging, biological aging, epigenetic clock, epigenetics

A hat trick of new epigenetic clocks has recently been published by Horvath et al.: The Skin & Blood clock [1] provides a more precise estimation of chronological age in tissues and cell types frequently used in research and forensics, while PhenoAge [2] and GrimAge [3] aim to capture biological aging and derive an improved prediction of mortality and morbidity risks. Together, these new epigenetic clocks present valuable tools to investigate human aging, shed light on the question of why we all age differently, and develop strategies to extend human life- and healthspan.

In 2013, the first epigenetic age estimation method that works with high accuracy across almost all human tissues and cell types was published by Steve Horvath [4]. The publication of this multi-tissue clock marked a milestone in epigenetics and aging research, and since then, numerous studies have confirmed not only its ability to accurately estimate an individual’s age but also the clock’s great value for studying the human aging process.

Horvath’s multi-tissue clock is based on DNA methylation data. DNA methylation, the addition of methyl groups to cytosine bases of the DNA, is the most widely studied epigenetic modification so far. It plays an important role in the regulation of gene expression, altering the phenotype without changing the genotype. A particular locus in the genome can either be methylated or unmethylated. But as DNA methylation measurements are usually obtained from a pool of tens of thousands of cells, what is measured, is the proportion of the cells in which a locus is methylated. These proportions are given in *β‑*values between 0 (unmethylated in all cells) and 1 (methylated in all cells). Thus, methylation *β‑*values effectively measure cell-to-cell variability within a sample.

In many positions of the human genome, this methylation heterogeneity changes with age. These usually small but consistent age-associated changes in DNA methylation are what make the epigenetic clock work. And it works very precisely, with a median absolute error (MAE) of only 3.6 years, clearly outperforming previously used molecular biomarkers of age such as telomere length [4,5].

However, deviations of the age estimation derived by DNA methylation compared to chronological age do also provide valuable information. There is significant interindividual variability present in the human aging process [6–8]. Biological aging occurs at different rates across individuals who can exhibit considerably distinct physical fitness and age-related disease susceptibilities despite being the same chronological age. The epigenetic clock has intriguingly demonstrated to be able to quantify these differences and give a biologically relevant prediction of age, that is, a measurement of biological or physiological age. DNA methylation age predicts all-cause mortality better than chronological age, and it has also been associated with physical and mental fitness, vegetable and fish intake, obesity, smoking, alcohol use, lifetime stress, social class and multiple other factors [5,9].

Recently, three further improved epigenetic clocks were published: The Skin & Blood clock [1], DNA methylation PhenoAge [2], and DNA methylation GrimAge [3]. While the latter two aim to provide an improved prediction of mortality and are more closely related to physiological dysregulation, the Skin & Blood clock gives an even more accurate prediction of chronological age of easily accessible human tissues – for example, whole blood, saliva and skin – and cell types often used in research such as fibroblasts and lymphoblastoid cell lines.

In a set of whole blood samples, the application of the Skin & Blood clock resulted in age estimations with a MAE of 2.5 [1]. The correlation between chronological age and estimated age also improved correspondingly, from a Pearson’s *r* of 0.96 for the multi-tissue clock to 0.98 in the Skin & Blood clock [1].

**Figure 1:**
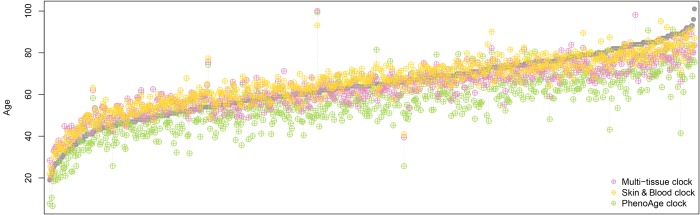
**Comparison of epigenetic age estimations derived by three different epigenetic clocks.** Each grey data point represents an individual whole blood sample, ordered by the chronological age of the sample donor from young to old. The coloured symbols show the epigenetic age estimation provided by the three clocks, where yellow represents the Skin & Blood clock, magenta the multi-tissue clock, and green the PhenoAge clock. The Skin & Blood clock’s age estimations come closest to the corresponding chronological ages. However, as the multi-tissue clock, it tends to show slightly increased epigenetic ages in younger individuals and decreased epigenetic ages in the elderly, while the PhenoAge clock predicts younger ages (in terms of life- and healthspan) for most individuals in the dataset used here [10]

We observed similar results when we applied the Skin & Blood clock, the multi-tissue clock, and the PhenoAge clock to a dataset of 656 samples (age range 19 to 101 years) with which another highly accurate epigenetic age estimator for whole blood samples was developed by Hannum et al. [10] [of note, we did not include GrimAge in these comparisons, as GrimAge is supposed to be a predictor of mortality rather than an age predictor and takes (chronological) age itself as input, together with sex and DNA methylation measurements]. The Skin & Blood clock exhibited the least error and the best correlation with chronological age (see Table 1 and Figure 1), confirming its improved performance in chronological age prediction.

**Table 1:** Comparison of the epigenetic age estimation of three different clocks.

**Clock**	**Pearson’s *r***	***p*-value**	**Median error**	**Maximum error**
**Skin & Blood**	0.94	5.03E-308	3.83	32.20
**Multi-tissue**	0.92	1.58E-259	3.84	39.08
**PhenoAge**	0.85	5.10E-186	7.88	47.56

Errors given in absolute numbers.

The high sensitivity of the Skin & Blood clock in the above-mentioned cell types makes it particularly interesting for *ex vivo* experiments, as it provides a critical advantage over the multi-tissue clock which does not work well in fibroblasts and epithelial cells, and can track the dynamic aging of cells in culture [1,4].

Age estimations closer to the individuals’ chronological ages could nonetheless also lead to decreased possibilities for the biomarker to capture biological aging. However, there is still considerable variation regarding the deviation from chronological age observable (Table 1 and Figure 1), and the Skin & Blood clock is highly predictive of time to death across multiple epidemiological cohort studies [1].

Notwithstanding, the best epigenetic mortality predictor reported so far is DNA methylation GrimAge [3], a composite biomarker based on DNA methylation surrogates for several blood plasma protein markers and smoking pack-years as well as chronological age and sex. This new epigenetic clock not only performs better in predicting time to death, time to coronary heart disease and time to cancer as compared to other epigenetic clocks but also shows associations with lifestyle factors and related variables that could not be observed with previous epigenetic clocks.

For example, a beneficial effect of physical exercise on GrimAge is reported. GrimAge also shows negative associations with vegetable and carbohydrate consumption and Omega-3 acid intake. Increased GrimAge – on the contrary – is associated with fat intake, insulin and glucose levels, and BMI and waist-to-hip ratio. Interestingly, GrimAge also shows a strong correlation with measures of adiposity derived by computed tomography, in particular regarding liver fat and visceral adipose tissue volume which are known to be detrimental to health. Altogether, these associations make DNA methylation GrimAge a promising biomarker for human health and physiological age.

While epigenetic clocks such as PhenoAge and GrimAge already provide valuable readouts of biological aging, the next challenge will be to develop epigenetic predictors that can distinguish between different reasons for increased (or decreased) mortality and morbidity. Specific risks for different diseases or disease types could be incorporated into assessments for individuals with altered epigenetic ages, possibly providing opportunities for better disease prevention.

Despite providing the most accurate molecular biomarkers of age to date, it remains a mystery why exactly the epigenetic clocks work, and whether age-related changes in DNA methylation contribute to the cause of aging or are a result of it. It has been hypothesized that the changes giving rise to DNA methylation age estimators are the molecular footprints of epigenetic developmental and maintenance programs, thus capturing at least a part of the biological aging mechanism [4–7]. This is an attractive hypothesis, as epigenetic changes are, in principle, reversible.

Epigenetic clocks have already proven to be an invaluable tool in determining the impact of different factors on aging. With their further developments, they provide an easily measurable outcome to evaluate and monitor interventions aimed to promote healthy aging, in both *in vitro* experiments and by tailoring lifestyles individually through personalized medicine and preventive health care [6,8]. How the latter can be implemented appropriately is a subject of intense debate. Enabled by its open consent and open access policy, the Personalized Genome Project UK [11] has pioneered epigenetic reports that inform participants of the project about their epigenetic ages. This can help participants to reflect on their lifestyles and implement changes to live and age healthier.

As epigenetic clocks are getting more and more accurate and informative, they also become promising candidates for applications in areas such as forensics, immigration and insurance. These new possibilities, however, come along with the potential for misuse and discrimination, and the current lack of regulation in this area needs to be urgently addressed.

Finally, without the open access movement, the development of epigenetic clocks would not have been possible (the first multi-tissue epigenetic clock was based on 8,000 openly available DNA methylation samples) [4,5], and with ever more data available, we will be able to continue to shed light on the complex phenotype of human aging. To this aim, collaboration across different disciplines will be indispensable, through the integration of electronic health records with detailed multi-omic data and deep phenotypes from longitudinal population cohorts and clinical studies. Epigenetic clocks will be key players in this process. And by providing insight into the question of why we all age differently and assisting the development and monitoring of strategies to extend life- and healthspan, the epigenetic clock may indeed become a molecular crystal ball for human aging.
